# Comprehensive analysis of pan‐cancer reveals potential of ASF1B as a prognostic and immunological biomarker

**DOI:** 10.1002/cam4.4203

**Published:** 2021-09-02

**Authors:** Xinyao Hu, Hua Zhu, Xiaoyu Zhang, Xiaoqin He, Ximing Xu

**Affiliations:** ^1^ Department of Oncology Renmin Hospital of Wuhan University Wuhan China; ^2^ Cancer Center Renmin Hospital of Wuhan University Wuhan China; ^3^ Department of Neurosurgery Renmin Hospital of Wuhan University Wuhan China

**Keywords:** ASF1B, MSI, pan‐cancer, prognosis, TMB, tumor immunity

## Abstract

**Background:**

Anti‐silencing function 1 (ASF1) is a conserved histone H3–H4 chaperone protein. ASF1B, a paralog of ASF1, acts by promoting cell proliferation and influencing cell cycle progression. Although there is some evidence demonstrating that ASF1B plays a key role in the development, progression, and prognosis of certain cancers, there are no pan‐cancer analyses of ASF1B.

**Methods:**

We used a range of bioinformatics approaches to investigate the predictive role of ASF1B, including its correlation with prognosis, tumor mutational burden (TMB), microsatellite instability (MSI), tumor microenvironment (TME), and immune cell infiltration, in diverse cancer types.

**Results:**

We found that ASF1B was highly expressed in 22 cancers and was negatively correlated with the prognosis of multiple major cancer types. Furthermore, ASF1B expression was correlated with TMB in 21 cancers and with MSI in 7 cancers. We found that ASF1B was coexpressed with genes encoding immune activators, immune suppressors, major histocompatibility complexes, chemokines, and chemokine receptors. We further found that the role of ASF1B in the infiltration of different types of immune cells varied across tumor types. ASF1B may potentially affect several key immune‐related pathways, such as those involved in antigen processing and presentation, natural killer cell‐mediated cytotoxicity, and autoimmune thyroid disease.

**Conclusions:**

Our findings show that ASF1B may serve as a prognostic marker and potential immunotherapeutic target for several malignancies due to its role in tumorigenesis and immune infiltration.

## Graphical abstract

Our first pan‐cancer analysis of ASF1B results showed that this gene was highly expressed in 22 tumor tissues compared to normal tissues and revealed an association between ASF1B expression and clinical prognosis. Our results also suggested that ASF1B may be an independent prognostic factor in multiple cancers and that high ASF1B expression is relatedto poor prognosis in major tumor types. Furthermore, ASF1B expression was associated with TMB, MSI, and immune cell infiltration in various cancers. Its effect on tumor immunity varied depending on the cancer type. These discoveriesmay contribute to clarifying the role of ASF1B in tumor development and provide new regulatory targets for more precise and personalized immune antitumor strategies.

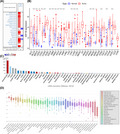

## 1 INTRODUCTION

Cancer is caused by a complicated series of events that leads to uncontrolled cell growth and enables cells to evade natural cell death mechanisms, resulting in malignancy and high lethality.^1^ Recent statistics indicated that cancer‐related mortality was expected to increase by 50% by 2020.^2^ Therefore, the identification of new prognostic and therapeutic biomarkers is urgent. In recent years, immunotherapy, especially immune checkpoint blockade therapy, has become a powerful clinical strategy for cancer treatment. The number of approved immunotherapy drugs, which kill cancer cells by activating and boosting the body's own immune system, continues to rise.^3^ With the development and refinement of pan‐cancer gene expression databases, it is easier than ever to identify new immunotherapeutic targets, assess their clinical value, and determine their associated signaling pathways.

Cancer is associated with aberrant gene expression and modification, processes in which histones play a crucial role. Anti‐silencing function 1 (ASF1), a conserved histone H3–H4 chaperone protein, regulates transcription by derepressing silenced mating‐type sites^4^, ^5^ and is expressed as two paralogs, ASF1A and ASF1B. ASF1A assists in DNA repair and promotes cellular senescence, while ASF1B is mainly involved in cell proliferation and cell cycle progression.^6^, ^7^ A previous study demonstrated that high ASF1B expression is highly correlated with prostate cancer (PCa) lymph node metastasis (TNM) staging and that silencing ASF1B inhibits the PI3K/Akt signaling pathway, thereby suppressing PCa cell proliferation and promoting apoptosis and cell cycle arrest.^8^ Similarly, ASF1B has been shown to be highly expressed in clear cell renal cell carcinoma (ccRCC), where its expression is dependent on the AKT/P70 S6K1 pathway and is correlated with tumor stage, tumor grade, and patient prognosis.^9^ It is worth noting that S6K1 was found to affect the expression of immune response genes.^10^ High ASF1B expression levels are also associated with disease progression and prognosis in lung adenocarcinoma (LUAD), breast cancer (BRCA), cervical cancer (CESC), and multiple myeloma (MM).^4^, ^11^, ^12^ Overall, ASF1B is gaining attention as an important player in the development of several tumors and is expected to become a new diagnostic and prognostic biomarker as well as a therapeutic target for these cancers. However, the role of ASF1B in other cancer types remains uncertain.

Bidirectional communication between the microenvironment and cells is essential for both tumor growth and normal tissue homeostasis.^13^ In addition to cancer cells, the tumor microenvironment (TME) contains immune cells, stromal cells, endothelial cells, and cancer‐associated fibroblasts. Cancer cells evade immune surveillance and clearance through a series of mechanisms; thus, immunotherapy is a promising approach for cancer treatment. Unlike conventional chemotherapy, immunotherapy boasts higher specificity and fewer side effects, mainly due to its use of immune cells both inside and outside the TME to specifically identify and attack cancer cells.^14^ An increasing number of studies have illustrated the important clinical value of combining chemotherapy with immune checkpoint inhibitors (ICIs) at appropriate doses. For example, combination treatment using the cyclophosphamide and ipilimumab (CTLA‐4 blockade) has shown promising results in the treatment of two mouse tumor models.^15^, ^16^ However, immunotherapy studies for various cancers are still limited, and further investigations into more specific or generalized immune targets for cancer immunotherapy are still needed to improve patient prognosis and quality of life.

We conducted a pan‐cancer analysis of ASF1B using multiple databases, including Oncomine, HPA, CCLE, and TGCA, to characterize ASF1B expression and its correlation with prognosis, immune response, clinicopathology, and tumor microenvironment in diverse cancers. Our findings revealed that ASF1B is correlated with the development of multiple cancers, has the potential to be a good diagnostic, therapeutic and prognostic marker, and is promising as a new immune checkpoint inhibitor. This work offers a basis for understanding the mechanism of action of ASF1B in various cancers and provides a rationale for targeting ASF1B with immunotherapies.

## 2 METHODS

### 2.1 Data processing and differential expression analysis

Oncomine (https://www.oncomine.org/), an online cancer microarray database, contains approximately 48 million gene expression data points from over 80,000 samples of various cancer types.^17^ We used this database to analyze the mRNA expression of ASF1B in 33 types of human malignancies. Filters were set as follows: gene symbol: “ASF1B,” datatype: “mRNA,” and cancer versus normal analysis. Thresholds included gene rank: 10%, fold change: 1.5, and *p*‐value: 0.001. Data sets with statistical significance were noted.

We downloaded 33 cancer‐related RNA sequencing datasets and their associated clinicopathological and survival data from the UCSC Xena website (https://xena.ucsc.edu/, derived from the TCGA). We then extracted ASF1B expression data from TCGA (https://tcga.xenahubs.net) using Perl software and performed pan‐cancer analyses. The “wilcox.test” method was applied to investigate ASF1B mRNA expression levels across cancers. Thereafter, we investigated mRNA sequencing in different cancer cell lines from Cancer Cell Line Encyclopedia (CCLE, https://portals.broadinstitute.org/ccle). The cutoff was set as a false discovery rate (FDR) value <0.05. The R package “ggpubr” was used to design the box plot.

### 2.2 Identification of the correlation between ASF1B expression levels and clinicopathology or survival in human cancers

We extracted the survival information for each TCGA sample. We then selected several indicators (overall survival [OS], disease‐specific survival [DSS], disease‐free interval [DFI], and progression‐free interval [PFI]) to investigate the association of ASF1B expression with the prognosis of patients with various cancers. We used the Kaplan–Meier method and log‐rank test to perform survival analyses in 33 cancer types (*p* < 0.05) and then plotted survival curves using the R packages “survminer” and “survival.” The R packages “survival” and “forestplot” were used for Cox analysis to identify the correlation between ASF1B and survival. The R packages “ggpubr” and “limma” were used for clinicopathological correlation analyses.

### 2.3 Association between ASF1B expression and tumor mutational burden (TMB) or microsatellite instability (MSI) across cancers

To calculate the number of somatic mutations in 33 cancers, TMB was evaluated based on Perl scripts and this value was corrected by dividing by the exon length. MSI scores were extracted using TCGA. The correlation between ASF1B expression and either TMB or MSI was analyzed using the “cor.test” command based on Spearman's method. The two metrics were visualized using radar plots, which were generating using the R package “fmsb.”

### 2.4 Association between ASF1B expression and the tumor immune microenvironment or tumor immune cell infiltration

TIMER (Tumor Immunization Estimation Resource) database (https://cistrome.shinyapps.io/timer/), a web server for comprehensive analysis of the infiltration of tumor immune cells, was used to explore the relationship between prognosis and the infiltration of tumor immune cells, including CD4^+^ T cells, CD8^+^ T cells, B cells, neutrophils, dendritic cells (DCs), and macrophages. We then explored the relationship between ASF1B expression levels and the abundance of tumor‐infiltrating immune cells in 15 cancers, in which ASF1B had a correlation with overall survival. We next applied the ESTIMATE algorithm from the R packages “estimate” and “limma” to calculate immune and stromal scores.^18^ We analyzed tumor purity and the infiltration of stromal/immune cells into the tumor tissue of various tumor types (*n* = 33) based on ASF1B expression data using CIBERSORT, which was developed to estimate the abundance of particular cells in hybrid cell populations using gene expression datasets.^19^ We next analyzed the correlation of ASF1B with the TME or immune cell infiltration using the R packages “ggplot2,” “ggpubr,” and “ggExtra” (with a cutoff value of *p* < 0.001).

### 2.5 Coexpression of ASF1B with immune‐related genes and pathways in tumors

The R packages “limma,” “reshape2,” and “RColorBrewer” were used for coexpression analyses. Gene ontology (GO) and Kyoto Encyclopedia of Genes and Genomes (KEGG) gene sets were obtained from the Gene Set Enrichment Analysis website (GSEA, https://www.gsea‐msigdb.org/gsea/downloads.jsp). GO and KEGG functional annotations and enriched pathways associated with ASF1B were analyzed using the R packages “limma,” “org. Hs.eg.db,” “clusterProfiler,”^20^ and “enrichplot.”

### 2.6 Statistical analysis

All gene expression data were subjected to log2 transformative normalization. Comparisons between normal and cancerous tissues were evaluated using two‐group *t*‐tests. Kaplan–Meier analyses, Cox proportional hazards models, and log‐rank tests were conducted for all survival analyses in our work. Correlations between two variables were analyzed using Spearman's test or Pearson's test; *p* < 0.05 was defined as a significant difference. All statistical analyses were conducted using R software (version 4.0.2).

## 3 RESULTS

### 3.1 Different ASF1B expression levels in tumor and normal tissues

**FIGURE 1:**
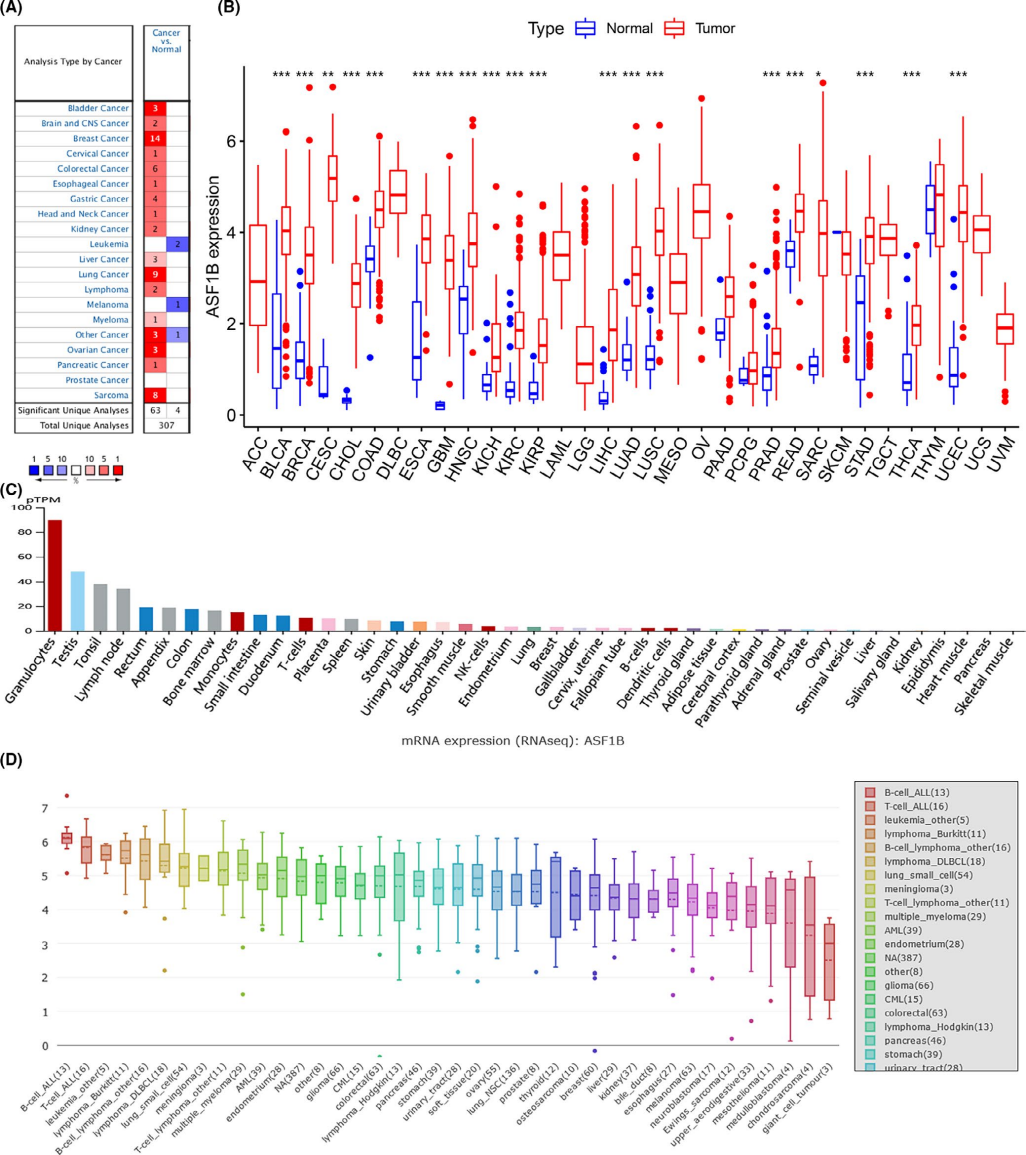
ASF1B expression levels in normal and tumor tissues. (A) ASF1B is upregulated in major tumor tissues compared to normal tissues. (B) ASF1B is differentially expressed in various tumor and normal tissues. (C) ASF1B is differentially expressed in various organs. (D) ASF1B mRNA expression levels in diverse cell lines. *p < 0.05, **p < 0.01, ***p < 0.001

We investigated ASF1B expression levels in normal tissues and various cancer tissues using Oncomine. The results indicated that the expression of ASF1B was significantly increased in most cancer types, including bladder cancer (BLCA), brain and central nervous system (CNS) cancer, BRCA, cervical cancer (CESC), colorectal cancer (COAD), esophageal cancer (ESCA), gastric cancer, head, and neck cancer (HNSC), kidney cancer, liver cancer (LIHC), lung cancer, lymphoma, myeloma, ovarian cancer (OV), pancreatic cancer (PAAD), and sarcoma (SARC) (Figure 1A). In contrast, lower expression of ASF1B was observed in several other cancers, including skin cutaneous melanoma (SKCM) (Figure 1A). These differences may be a result of different data collection methods or different biological mechanisms of tumor development.

To further assess ASF1B expression across cancers, we used R software to analyze RNA sequencing data obtained from TCGA (Figure 1B). A total of 11,057 TCGA profiles (included 730 normal and 10,327 tumor samples) of mRNA expression for 33 cancers were gained. Table S1 shows the amount of different cancer and normal samples contained in this study. We found that ASF1B expression was significantly elevated in 20 of the 33 cancer types, including BLCA, BRCA, CESC, bile duct cancer (CHOL), COAD, ESCA, glioblastoma (GBM), HNSC, kidney chromophobe (KICH), kidney clear cell carcinoma (KIRC), kidney papillary cell carcinoma (KIRP), LIHC, LUAD, lung squamous cell carcinoma (LUSC), prostate cancer (PRAD), rectal cancer (READ), SARC, stomach cancer (STAD), thyroid cancer (THCA), and endometrioid cancer (UCEC). We did not observe lower ASF1B expression in any of the 33 cancers relative to normal tissues. Notably, there was no significant difference in ASF1B expression between PAAD and normal tissues, pheochromocytoma & paraganglioma (PCPG), and normal tissues, or thymoma (THYM) and normal tissues. ASF1B expression was significantly elevated in some cancers with only a few available normal samples, such as diffuse large B‐cell lymphoma (DLBC) and ovarian cancer (OV). This indicated that the expression of ASF1B in normal tissues should be studied using other databases. Therefore, we conducted an additional investigation using the HPA database. We discovered that ASF1B was highly expressed in granulocytes, tonsils, and lymph nodes, all of which play important roles in the immune system (Figure 1C). We also investigated ASF1B mRNA expression levels in 33 cancer tissues using the CCLE database and found that ASF1B mRNA was highly expressed in B‐cell acute lymphoblastic leukemia (ALL), T‐cell ALL, other leukemias, Burkitt lymphoma, other B‐cell lymphomas, and diffuse large B‐cell lymphoma (DUBCL) (Figure 1D). We also found that ASF1B was expressed at low levels in the ovary and skin (Figure 1C) but was highly expressed in OV and SKCM (Figure 1B). Overall, our results indicated that ASF1B expression was significantly higher in 22 cancer types relative to normal tissues.

### 3.2 Pan‐cancer prognostic value of ASF1B

**FIGURE 2:**
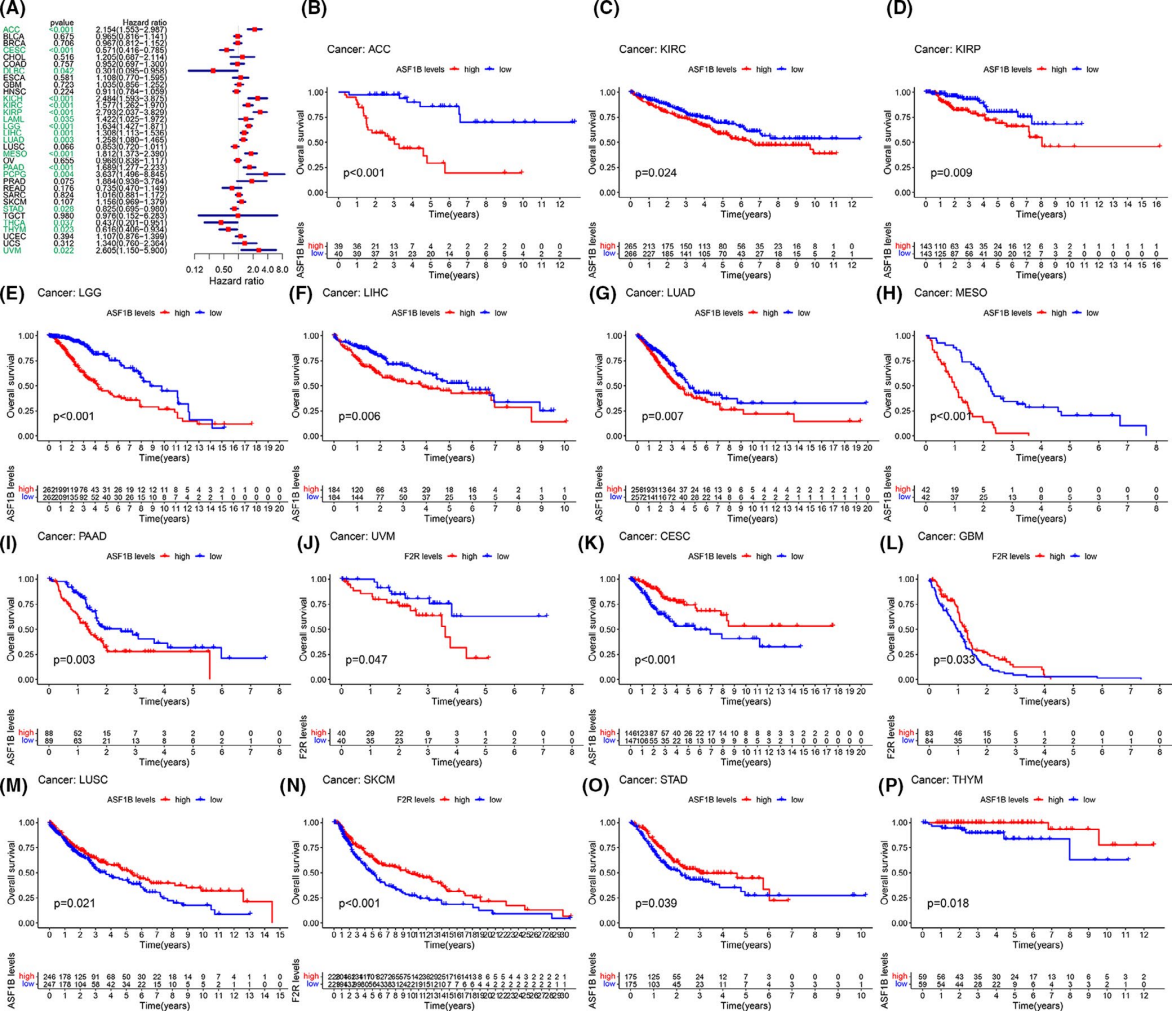
ASF1B expression correlated with overall survival time (OS). (A) Forest plots showing correlations between OS and ASF1B expression in 33 cancer types. Kaplan‐Meier analyses of the association between ASF1B expression and OS in (B) adrenocortical carcinoma (ACC), (C) kidney clear cell carcinoma (KIRC), (D) kidney papillary cell carcinoma (KIRP), (E) lower grade glioma (LGG), (F) liver cancer (LIHC), (G) lung adenocarcinoma (LUAD), (H) mesothelioma (MESO), (I) pancreatic cancer (PAAD), (J) uveal melanoma (UVM), (K) cervical cancer (CESC), (L) cervical cancer (GBM), (M) lung squamous cell carcinoma (LUSC), (N) skin cutaneous melanoma (SKCM), (O) stomach cancer (STAD), (P) thymoma (THYM)

To further investigate the correlation between ASF1B expression and prognosis, we conducted survival correlation analyses in 33 cancers using the following metrics: overall survival (OS), disease‐free survival (DSS), disease‐free interval (DFI), and progression‐free interval (PFI). Cox analysis showed that ASF1B expression was significantly correlated with OS in ACC (*p* < 0.001), CESC (*p* < 0.001), DLBC (*p* = 0.042), KICH (*p* < 0.001), KIRC (*p* < 0.001), KIRP (*p* < 0.001), acute myeloid leukemia (LAML) (*p* = 0.035), lower grade glioma (LGG) (*p* < 0.001), LIHC (*p* = 0.001), LUAD (*p* = 0.003), mesothelioma (MESO) (*p* < 0.001), PAAD (*p* < 0.001), PCPG (*p* = 0.004), STAD (*p* = 0.028), THCA (*p* = 0.037), THYM (*p* = 0.023), and uveal melanoma (UVM) (*p* =< 0.022) (Figure 2A). Our results demonstrated that ASF1B expression was a high‐risk indicator in ACC, CESC, DLBC, KICH, KIRC, KIRP, LAML, LGG, LIHC, LUAD, MESO, PAAD, PCPG, STAD, THCA, THYM, and UVM, particularly PCPG (hazard ratio = 3.637). Conversely, ASF1B expression was an indicator of low risk in DLBC. Additionally, Kaplan–Meier analyses indicated that ACC (Figure 2B, *p* < 0.001), KIRC (Figure 2C, *p* = 0.024), KIRP (Figure 2D, *p* = 0.009), LGG (Figure 2E, *p* < 0.001), LIHC (Figure 2F, *p* = 0.006), LUAD (Figure 2G, *p* = 0.007), MESO (Figure 2H, *p* < 0.001), PAAD (Figure 2I, *p* = 0.003), and UVM (Figure 2J, *p* = 0.047) patients with high ASF1B expression displayed decreased survival. However, among the individuals with CESC (Figure 2K, *p* < 0.001), GBM (Figure 2L, *p* = 0.033), LUSC (Figure 2M, *p* = 0.021), SKCM (Figure 2N, *p* < 0.001), STAD (Figure 2O, *p* = 0.039), and THYM (Figure 2P, *p* = 0.018), those with high ASF1B expression had increased survival.

**FIGURE 3:**
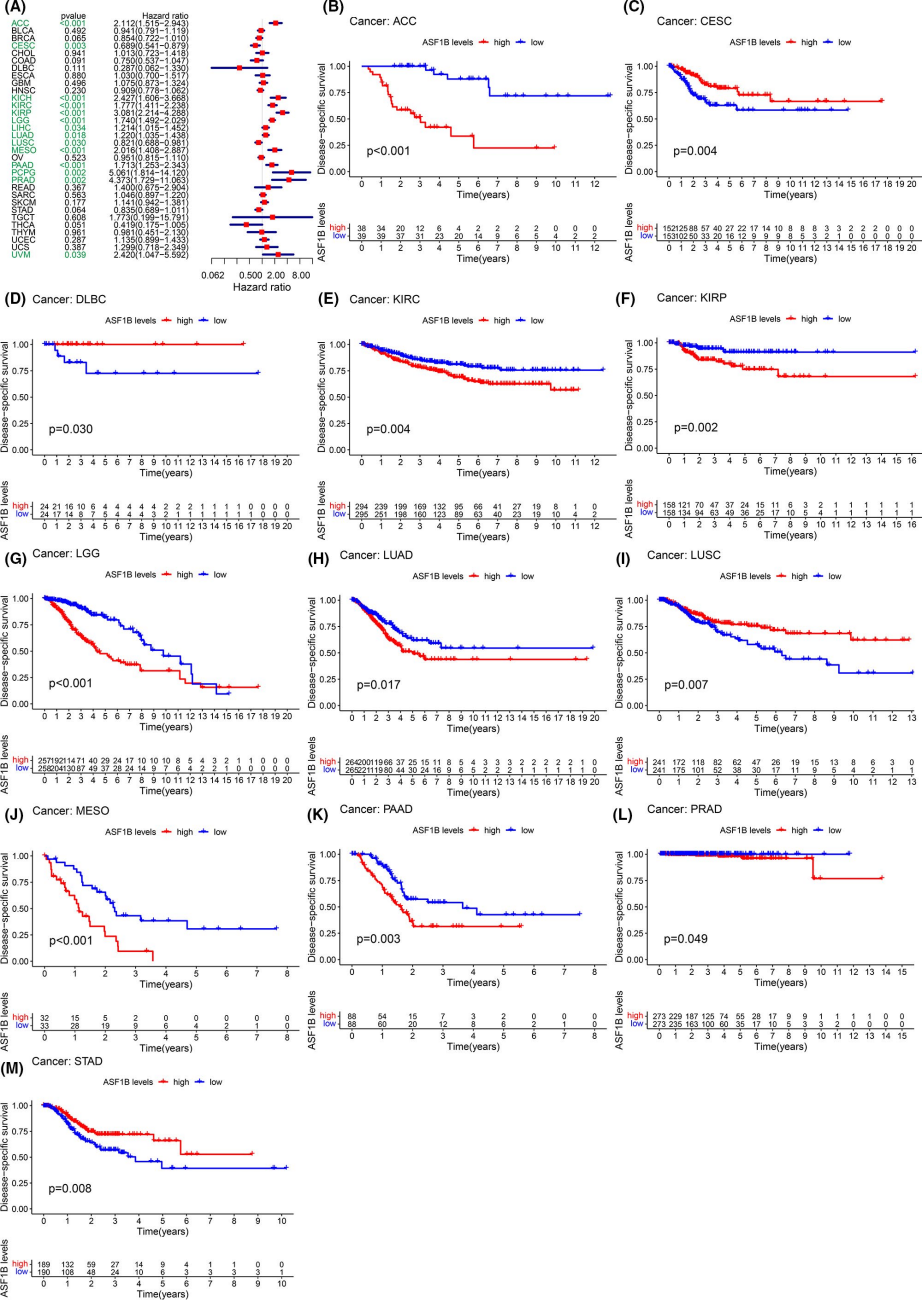
Correlation between ASF1B expression and disease‐specific survival (DSS). (A) Forest plots showing correlations between DSS and ASF1B expression in 33 tumor types. Kaplan‐Meier analyses of the association between ASF1B expression and DSS in (B) adrenocortical carcinoma (ACC), (C) cervical cancer (CESC), (D) diffuse large B‐cell lymphoma (DLBC), (E) kidney clear cell carcinoma (KIRC), (F) kidney papillary cell carcinoma (KIRP), (G) lower grade glioma (LGG), (H) lung adenocarcinoma (LUAD), (I) lung squamous cell carcinoma (LUSC), (J) mesothelioma (MESO), (K) pancreatic cancer (PAAD), (L) prostate cancer (PRAD), (M) stomach cancer (STAD)

**FIGURE 4:**
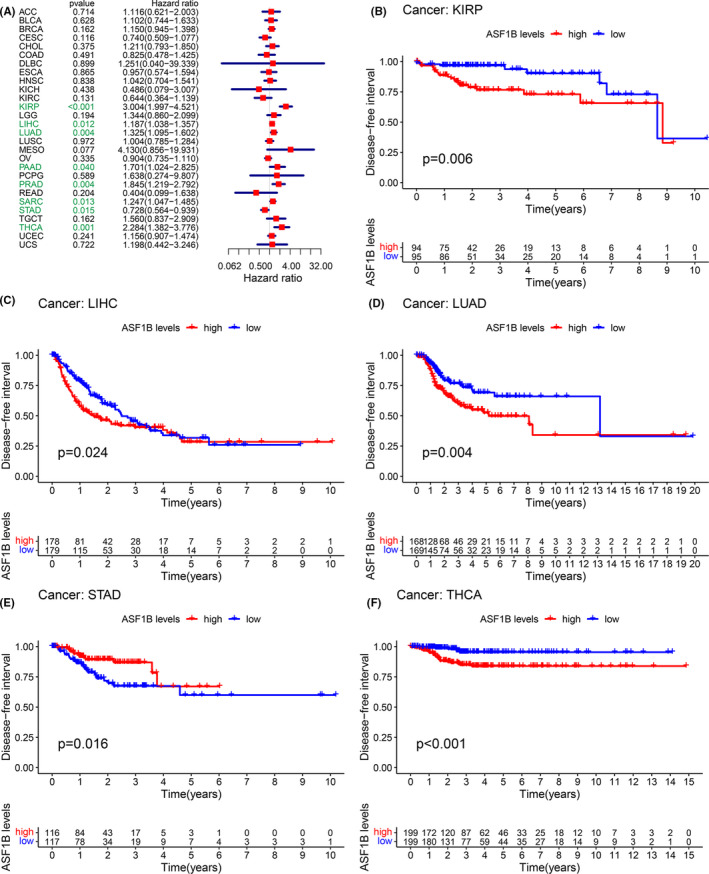
Correlation between ASF1B expression and disease‐free interval (DFI). (A) Forest plots showing the relationship between ASF1B expression and DFI in 33 tumor types. Kaplan‐Meier analyses of the association between ASF1B expression and DFI in (B) kidney papillary cell carcinoma (KIRP), (C) liver cancer (LIHC), (D) lung adenocarcinoma (LUAD), (E) stomach cancer (STAD), (F) thyroid cancer (THCA)

**FIGURE 5:**
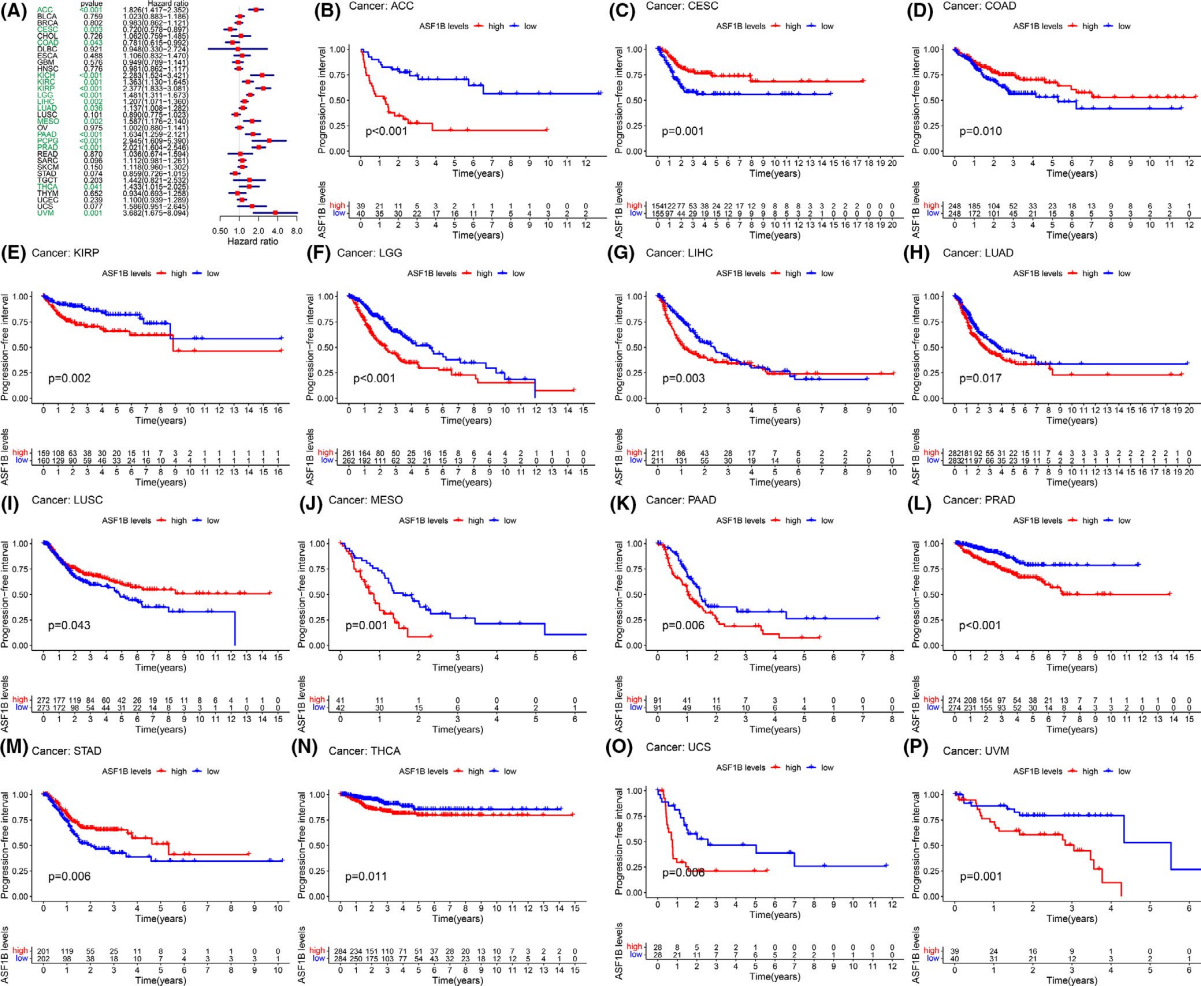
Correlation between ASF1B expression and disease‐free interval (DFI). (A) Forest plots showing correlations between ASF1B expression and DFI in 33 tumor types. Kaplan‐Meier analyses of the association between ASF1B expression and DFI in (B) adrenocortical carcinoma (ACC), (C) cervical cancer (CESC), (D) colon adenocarcinoma (COAD), (E) kidney papillary cell carcinoma (KIRP), (F) lower grade glioma (LGG), (G) liver cancer (LIHC), (H) lung adenocarcinoma (LUAD), (I) lung squamous cell carcinoma (LUSC), (J) mesothelioma (MESO), (K) pancreatic cancer (PAAD), (L) prostate cancer (PRAD), (M) stomach cancer (STAD), (N) thyroid cancer (THCA), (O) uterine carcinosarcoma (UCS), (P) uveal melanoma (UVM)

Moreover, DSS analyses showed that high ASF1B expression predicted adverse outcomes in individuals (Figure 3A) with ACC (*p* < 0.001), CESC (*p* = 0.003), KICH (*p* < 0.001), KIRC (*p* < 0.001), KIRP (*p* < 0.001), LGG (*p* < 0.001), LIHC (*p* = 0.034), LUAD (*p* = 0.0018), LUSC (*p* = 0.030), MESO (*p* < 0.001), PAAD (*p* < 0.001), PCPG (*p* = 0.002), PRAD (*p* = 0.002), and UVM (*p* = 0.0039). In these analyses, PCPG showed the highest hazard ratio (HR = 5.061). Kaplan–Meier analyses also indicated that high ASF1B expression was correlated with decreased DSS in patients with ACC (Figure 3B, *p* < 0.001), KIRC (Figure 3E, *p* = 0.004), KIRP (Figure 3F, *p* = 0.002), LGG (Figure 3G, *p* < 0.001), LUAD (Figure 3H, *p* = 0.017), MESO (Figure 3J, *p* < 0.001), PAAD (Figure 3K, *p* = 0.003), and PRAD (Figure 3L, *p* = 0.049). However, this association was reversed in CESC (Figure 3C, *p* = 0.004), DLBC (Figure 3D, *p* < 0.030), LUSC (Figure 3I, *p* = 0.007), and STAD (Figure 3M, *p* = 0.008). We further detected a correlation between high ASF1B expression and decreased DFI (Figure 4A) in KIRP (*p* < 0.001), LIHC (*p* = 0.012), LUAD (*p* = 0.004), PAAD (*p* = 0.004), PRAD (*p* < 0.001), SARC (*p* = 0.013), STAD (*p* = 0.015), and THCA (*p* = 0.001). Kaplan–Meier analyses also indicated that high ASF1B expression was associated with diminished DFI in individuals with KIRP (Figure 4B, *p* = 0.006), LIHC (Figure 4C, *p* = 0.024), LUAD (Figure 4D, *p* = 0.004), and THCA (Figure 4F, *p* < 0.001). Conversely, increased ASF1B expression was predictive of increased DFI in STAD (Figure 4E, *p* = 0.016). Forest plots indicated that high ASF1B expression was correlated with lower PFI in ACC (*p* < 0.001), CESC (*p* = 0.003), COAD (*p* = 0.043), KICH (*p* < 0.001), KIRC (*p* = 0.001), KIRP (*p* < 0.001), LGG (*p* < 0.001), LIHC (*p* = 0.002), LUAD (*p* = 0.036), MESO (*p* = 0.002), PAAD (*p* < 0.001) PCPG (*p* < 0.001), PRAD (*p* < 0.001), THCA (*p* = 0.041), and UVM (*p* = 0.001) (Figure 5A). KM survival analyses also revealed a significant correlation between ASF1B expression and decreased PFI in ACC (Figure 5B, *p* < 0.001), KIRP (Figure 5E, *p* = 0.002), LGG (Figure 5F, *p* < 0.001), LUAD (Figure 5H, *p* = 0.017), MESO (Figure 5J, *p* = 0.001), PAAD (Figure 5K, *p* = 0.006), and THCA (Figure 5N, *p* = 0.011). Meanwhile, increased ASF1B expression was correlated with increased PFI in individuals with CESC (Figure 5C, *p* = 0.001), COAD (Figure 5D, *p* = 0.010), LUSC (Figure 5I, *p* = 0.043), and STAD (Figure 5M, *p* = 0.006). Interestingly, patients with LIHC (Figure 5G, *p* = 0.003) showed decreased PFI in early disease stages but increased PFI in late disease stages, while the opposite trend was observed in individuals with uterine carcinosarcoma UCS (Figure 5O, *p* = 0.006).

### 3.3 Correlations between ASF1B expression and pan‐cancer clinicopathology

**FIGURE 6:**
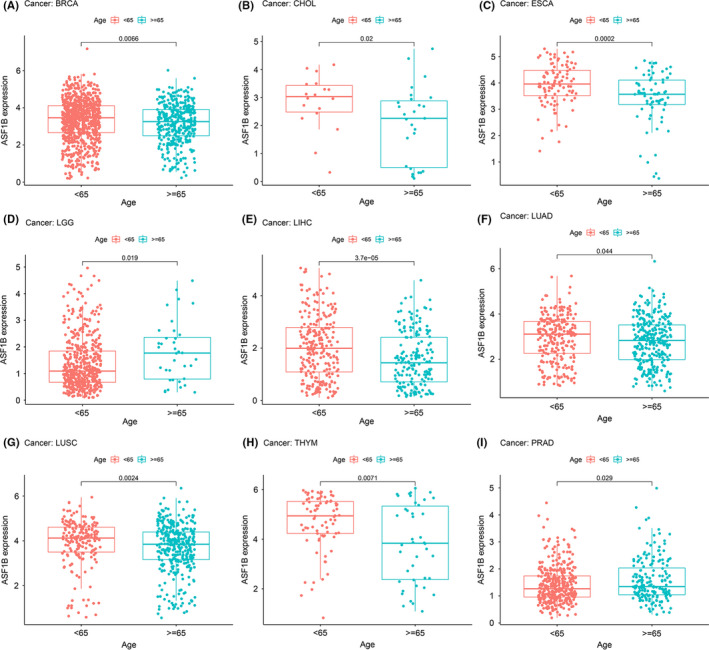
Relationship between ASF1B expression and age in (A) breast cancer (BRCA), (B) bile duct cancer (CHOL), (C) esophageal cancer (ESCA), (D) lower‐grade glioma (LGG), (E) liver cancer (LIHC), (F) lung adenocarcinoma (LUAD), (G) lung squamous cell carcinoma (LUSC), (H) thymoma (THYM), and (I) prostate cancer (PRAD)

We next evaluated differences in ASF1B expression based on age in patients with various types of tumors. Our results indicated that patients aged over 65 years with BRCA (Figure 6A, *p* = 0.0066), CHOL (Figure 6B, *p* = 0.02), ESCA (Figure 6C, *p* = 0.0002), LIHC (Figure 6E, *p* = 3.7e‐05), LUAD (Figure 6F, *p* = 0.044), LUSC (Figure 6G, *p* = 0.0024), and THYM (Figure 6H, *p* = 0.0071) had lower ASF1B expression. However, patients >65 years with LGG (Figure 6D, *p* = 0.019) and PRAD (Figure 6I, *p* = 0.029) had higher expression of ASF1B relative to patients <65 years. No prominent correlation between age and ASF1B expression was observed in patients with other cancer types (Figure S1).

**FIGURE 7:**
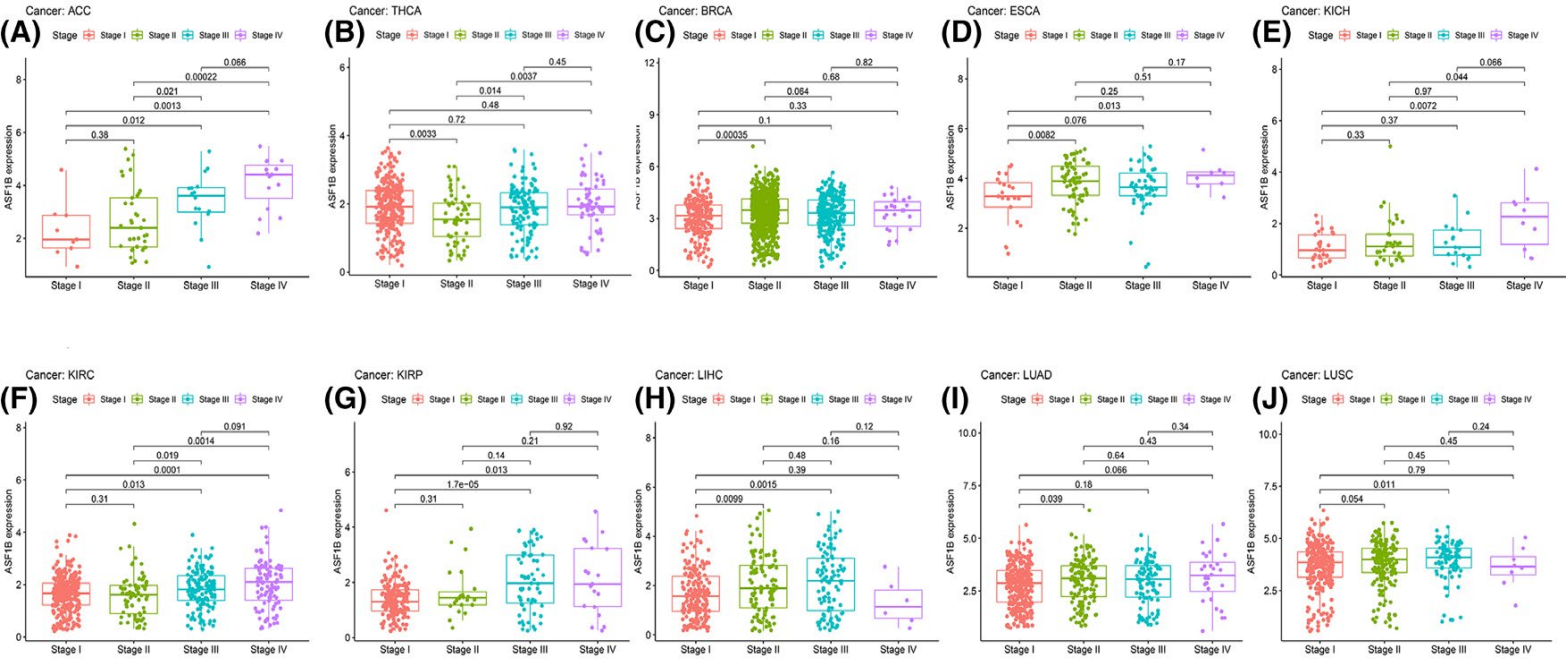
Correlation between ASF1B expression and tumor stage in (A) adrenocortical cancer (ACC), (B) bladder cancer (BLCA), (C) breast cancer (BRCA), (D) esophageal cancer (ESCA), (E) kidney chromophobe (KICH), (F) kidney clear cell carcinoma (KIRC), (G) kidney papillary cell carcinoma (KIRP), (I) lung adenocarcinoma (LUAD), and (J) lung squamous cell carcinoma (LUSC)

We then examined the association between ASF1B expression and tumor stage and discovered that ASF1B expression was dramatically correlated with tumor stage in 11 cancers, including ACC, BLCA, BRCA, ESCA, KICH, KIRC, KIRP, LIHC, LUAD, LUSC, and UVM (Figure S2). It is worth noting that the most significant differences in ASF1B expression existed mainly between stage I and stage IV cancers (Figure 7). ASF1B expression was notably increased in stage IV tumors compared with stage I tumors in patients with ACC (Figure 7A, *p* = 0.0013), ESCA (Figure 7D, *p* = 0.013), KICH (Figure 7E, *p* = 0.0072), KIRC (Figure 7F, *p* = 0.0001), KIRP (Figure 7G, *p* = 0.013), and LUAD (Figure 7I, *p* = 0.066). Moreover, in individuals with ACC (Figure 7A, *p* = 0.012), KIRC (Figure 7F, *p* = 0.013), KIRP (Figure 7G, *p* < 0.001), LIHC (Figure 7H, *p* = 0.0015), and LUSC (Figure 7J, *p* = 0.011), ASF1B expression was higher in stage III tumors than in stage I tumors. Intriguingly, in patients with THCA (Figure 7B, *p* = 0.0033) and BRCA (Figure 7C, *p* = 0.00035), ASF1B expression was higher in stage II tumors than stage I tumors but was not dramatically different in stage III or IV tumors compared to stage I tumors. Therefore, we surmised that, in patients with these advanced cancers, high ASF1B expression was directly responsible for decreased survival. Notably, although the ASF1B expression differences between stage I and IV tumors were remarkable, the expression differences between tumors of other stages were relatively small (Figure 7, Figure S2), and no statistically significant differences were found in other cancers (Figure S2).

### 3.4 Correlation between ASF1B expression and TMB or MSI in various cancers

**FIGURE 8:**
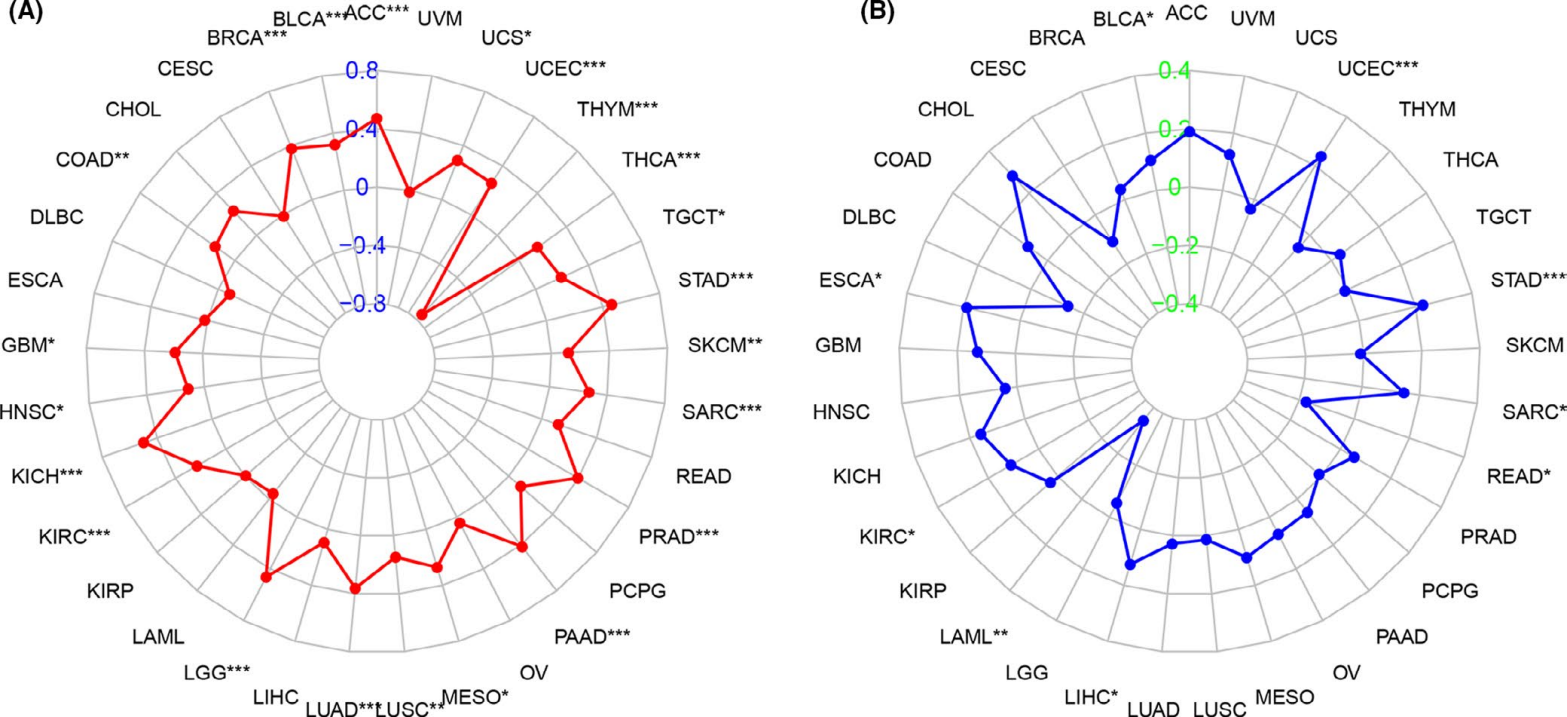
Correlation between ASF1B expression and tumor mutational burden (TMB) or microsatellite instability (MSI) in various cancer types. (A) Radar plot showing the correlation between ASF1B expression and TMB across all cancer types. Red lines represent correlation coefficients, and blue values represent ranges. (B) Radar plot showing the correlation between ASF1B expression and MSI across all cancer types. The blue lines represent correlation coefficients, and green values represent ranges

We then investigated the relationship between ASF1B expression and TMB and MSI, both of which are strongly associated with sensitivity to immune checkpoint inhibitors (ICIs) across cancers.^21^, ^22^ The results showed that ASF1B expression was associated with TMB in several cancers (*n* = 22, *p* < 0.05). Overall, the expression of ASF1B was positively correlated with TMB in 21 cancer types, including ACC, UCS, UCEC, THCA, TGCT, STAD, SKCM, SARC, PRAD, PAAD, MESO, LUSC, LUAD, LGG, KIRC, KICH, HNSC, GBM, COAD, BRCA, and BLCA (Table 1; Figure 8A), and negatively correlated with TMB in THYM (Table 1; Figure 8A). We further found that ASF1B expression was positively correlated with MSI in seven cancer types, including UCEC, STAD, SARC, LIHC, KIRC, ESCA, and BLCA (Table 1; Figure 8B), and negatively correlated with MSI in READ and LAML (Table 1; Figure 8B).

**TABLE 1:** Correlation of ASF1B expression with TMB, MSI

TMB			MSI		
Cancer type	Cor	*p*	Cancer type	Cor	*p*
ACC	0.47279769	***/0.00001084	BLCA	0.104068343	*/0.0356114
BLCA	0.31859024	***/4.46078E‐11	KIRC	0.109159884	*/0.045884341
BRCA	0.37737814	***/2.52036E‐34	LAML	−0.34296239	**/0.004195264
COAD	0.16425814	**/0.00103560	READ	−0.174985007	*/0.031066812
GBM	0.19020158	*/0.02058770	SARC	0.145937779	*/0.02022034
HNSC	0.11185389	*/0.01304556	STAD	0.226497893	***/9.72E‐06
KICH	0.49687743	***/0.00002552	UCEC	0.238371404	***/2.18E‐08
KIRC	0.22956412	***/0.00002404			
LGG	0.46341236	***/4.87452E‐28			
LUAD	0.36214289	***/4.93595E‐17			
LUSC	0.14759129	**/0.00107562			
MESO	0.27144051	*/0.01552992			
PAAD	0.41542294	***/0.00000011			
PRAD	0.39654722	***/1.33609E‐19			
SARC	0.27546200	***/0.00001841			
SKCM	0.11945224	**/0.00993293			
STAD	0.46294632	***/6.02813E‐21			
TGCT	0.19511429	*/0.01868271			
THCA	0.15653528	***/0.00056263			
THYM	−0.75058973	***/1.9865E‐22			
UCEC	0.25873463	***/1.777E‐09			
UCS	0.29036949	*/0.02993660			

*p < 0.05, **p < 0.01, ***p < 0.001.

### 3.5 Correlation of ASF1B expression with TME across cancers

**FIGURE 9:**
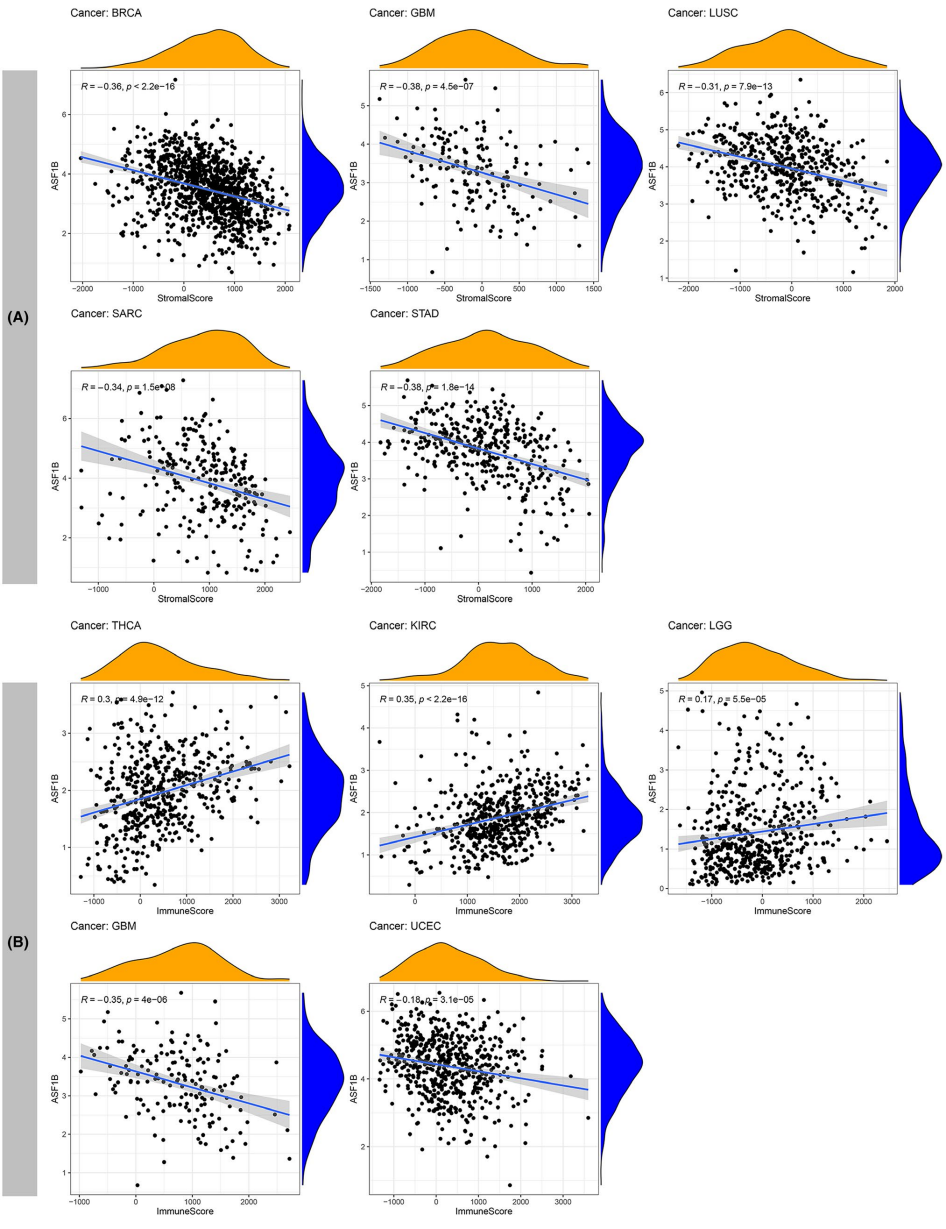
Five cancers with the highest association coefficients between ASF1B expression and the TME. (A) ASF1B expression was negatively associated with stromal scores in breast cancer (BRCA), glioblastoma (GBM), lung squamous cell carcinoma (LUSC), sarcoma (SARC) and stomach cancer (STAD). (B) ASF1B expression was negatively correlated with immune scores in GBM and endometrioid cancer (UCEC) and positively correlated with immune scores in thyroid cancer (THCA), kidney clear cell carcinoma (KIRC) and low grade glioma (LGG)

An increasing number of studies have revealed that the TME plays a crucial role in tumorigenesis and progression.^23^, ^24^ Genetic alterations in tumor cells lead to uncontrolled growth, resistance to apoptosis, and metabolic shifts toward anaerobic glycolysis (Warburg effect). These events trigger hypoxia, acidosis, and oxidative stress in the TME, initiate regulation of the extracellular matrix (ECM), elicit responses from adjacent immune cells (lymphocytes and macrophages), and stromal cells (e.g., fibroblasts), induce angiogenesis and eventually lead to cancer development and metastasis.^25^ Therefore, it is important to reveal the pan‐cancer associations between ASF1B expression and the TME. The ESTIMATE algorithm was used to assess stromal and immune scores for 33 cancers and to analyze their association with ASF1B expression. The findings indicated that ASF1B expression was negatively associated with stromal scores in BLCA, COAD, HNSC, LIHC, LUAD, OV, PAAD, and UCEC (Figure 9A; Figure S3). Additionally, the expression of ASF1B was significantly negatively correlated with immune scores in GBM and UCEC and positively correlated with immune scores in THCA, KIRC, and LGG (Figure 9B; Figure S3). No significant differences were found in other cancer types. The five cancer types with the highest association coefficients between the TME and ASF1B expression are presented in Figure 9; the results for other cancers are shown in Table S2 and Figure S3.

### 3.6 Association of ASF1B expression with immune cell infiltration in various cancers

**FIGURE 10:**
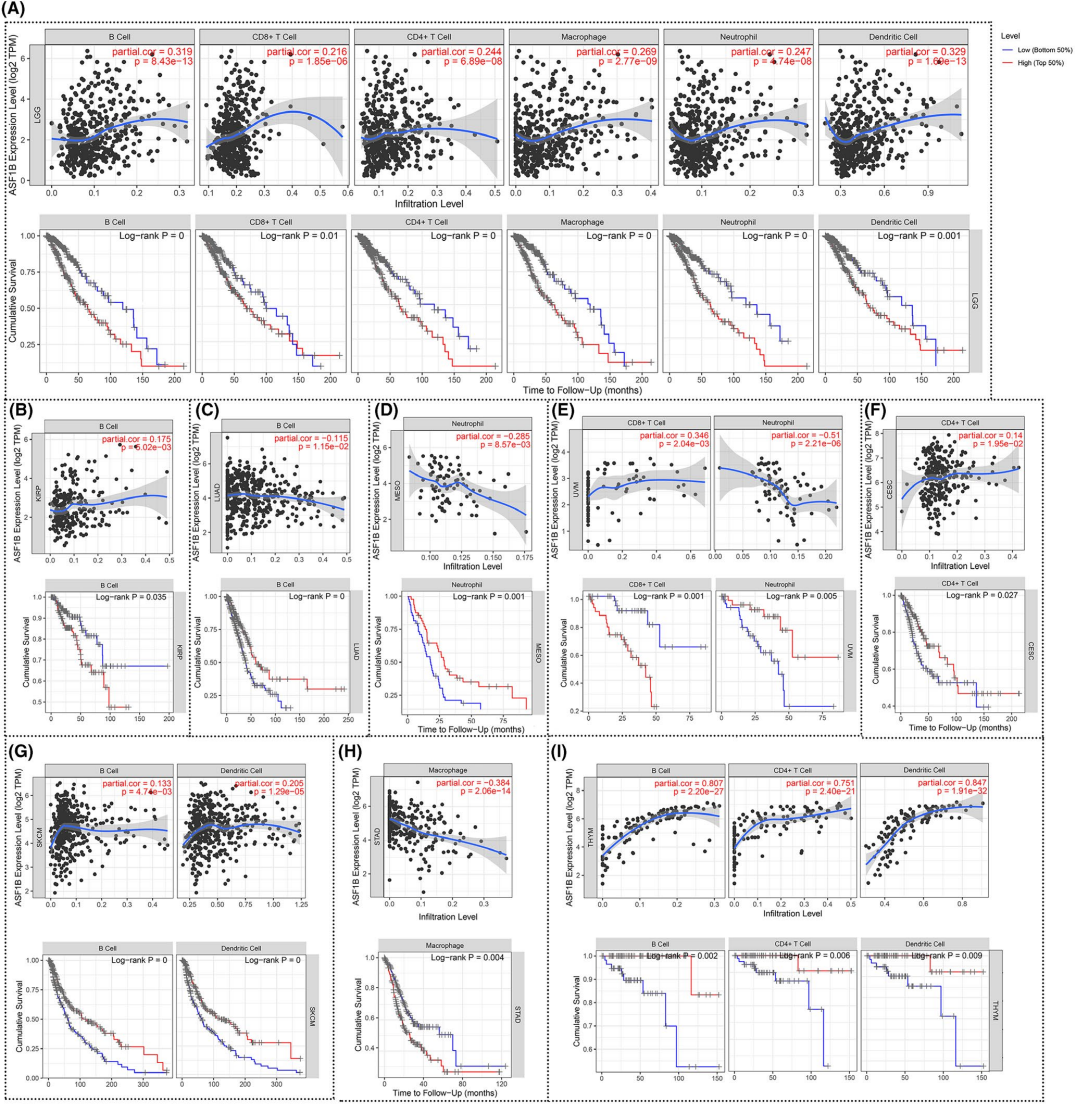
Association of ASF1B expression with infiltration levels of different immune cells, and correlation between immune cell infiltration and prognosis in (A) lower grade glioma (LGG), (B) kidney papillary cell carcinoma (KIRP), (C) lung adenocarcinoma (LUAD), (D) mesothelioma (MESO), (E) uveal melanoma (UVM), (F) cervical cancer (CESC), (G) skin cutaneous melanoma (SKCM), (H) stomach cancer (STAD), and (I) thymoma (THYM)

To further investigate the immune predictive value and immune correlation of ASF1B, we used TIMER to analyze the relationship among the ASF1B expression, the prognosis, and infiltration of six immune cells including B cell, CD8^+^ T cell, CD4^+^ T cell, macrophage, neutrophil, and DCs. We found that ASF1B was positively correlated with the infiltration of B cell (*p* < 0.001), CD8^+^ T cell (*p* < 0.001), CD4^+^ T cell (*p* < 0.001), macrophage (*p* < 0.001), neutrophil (*p* < 0.001), and DCs (*p* < 0.001) in LGG. Besides, high levels of B cells (*p* < 0.001), CD8^+^ T cells (*p* = 0.01), CD4^+^ T cell (*p* < 0.001), macrophage (*p* < 0.001), neutrophil (*p* < 0.001), and dendritic cell (*p* = 0.01) was related to poor prognosis of patients (Figure 10A). The combined analysis of the above two results implied that high ASF1B expression increased the infiltration levels of B cell, CD8^+^ T cell, CD4^+^ T cell, macrophage, neutrophil, and dendritic cell, leading to a poor prognosis in LGG, which was consistent with the previous results (Figure 2E). Additionally, in KIRP, ASF1B expression (Figure 10B) was positively correlated with B‐cell content (*p* = 0.005), which was negatively correlated with survival rate (*p* = 0.035), indicating that high expression of ASF1B predicted poor prognosis in patients with KIRP, which was in accordance with the results in Figure 2D. Inversely, high expression of ASF1B (Figure 10C) had a negative correlation with B cell infiltration in LUAD (*p* = 0.012), and higher B cell infiltration suggested a better prognosis (*p* < 0.001). Although the mechanisms were different, high levels of ASF1B also predicted poor prognosis of LUAD patients, which was compatible with our previous findings (Figure 2G). In MESO (Figure 10D), high ASF1B expression was negatively correlated with neutrophil infiltration (*p* = 0.009) and low levels of neutrophils in tumor tissue leaded to poor patient prognosis (*p* = 0.001). Hence, high expression of ASF1B was correlated with poor outcomes of patients with MESO (Figure 2H). Similarly, further analyses indicated that high ASF1B expression may decrease the infiltration of CD8^+^ T cell (*p* = 0.002) and increase the infiltration of neutrophil (*p* < 0.001) in UVM to reduce the survival time of patients with UVM (Figure 10E, Figure 2J). However, in CESC, SKCM, STAD, and THYM (Figure 10F–I), ASF1B expression may regulate the immune cell infiltration and had a positive correlation with the prognosis, which is also consistent with our results in Figure 2.

**FIGURE 11:**
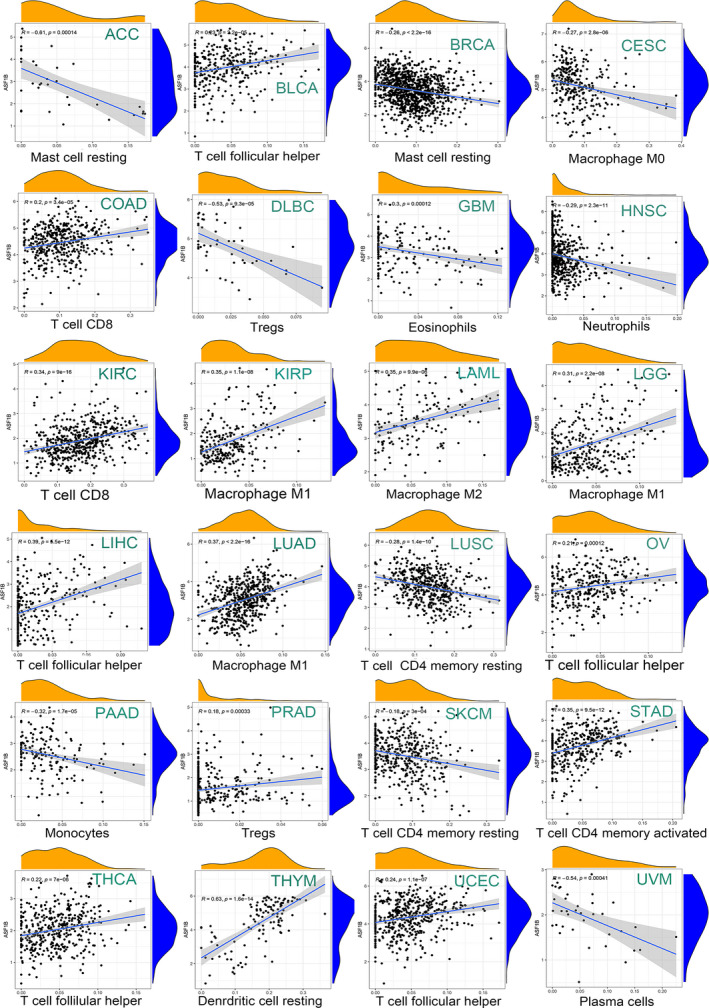
Association of ASF1B expression with infiltration levels of different immune cells in various cancers

We next examined the relationship between ASF1B expression and infiltration of 22 immune cell subtypes. The findings showed that immune cell infiltration levels were significantly correlated with the expression of ASF1B in most cancer types (Table S2). ASF1B expression was negatively correlated with levels of memory CD4 T cells (except in LUAD and SKCM) and regulatory T cells (Tregs) (except in HNSC, KIRC, and PRAD). However, ASF1B expression was positively associated with the levels of follicular helper T cells and CD8 T cells (Figure 11; Table S2). Moreover, ASF1B expression levels were associated with several different subpopulations of invasive macrophages. For instance, the expression of ASF1B was positively correlated with M0 macrophage infiltration in BRCA, LGG, LUAD, and STAD but negatively correlated with infiltration in CESC, HNSC, KIRP, and THYM. In BRCA, KIRP, LGG, LUAD, LUSC, STAD, and UCEC, ASF1B expression was positively associated with M1 macrophage levels. Furthermore, we observed a negative correlation between ASF1B expression and M2 macrophages in all cancer types except LAML (Figure 11; Table S2). Correlations between other immune cells and ASF1B expression are also presented in Figure 11.

### 3.7 Correlations between ASF1B expression and immune‐related genes and associated pathways in various cancers

**FIGURE 12:**
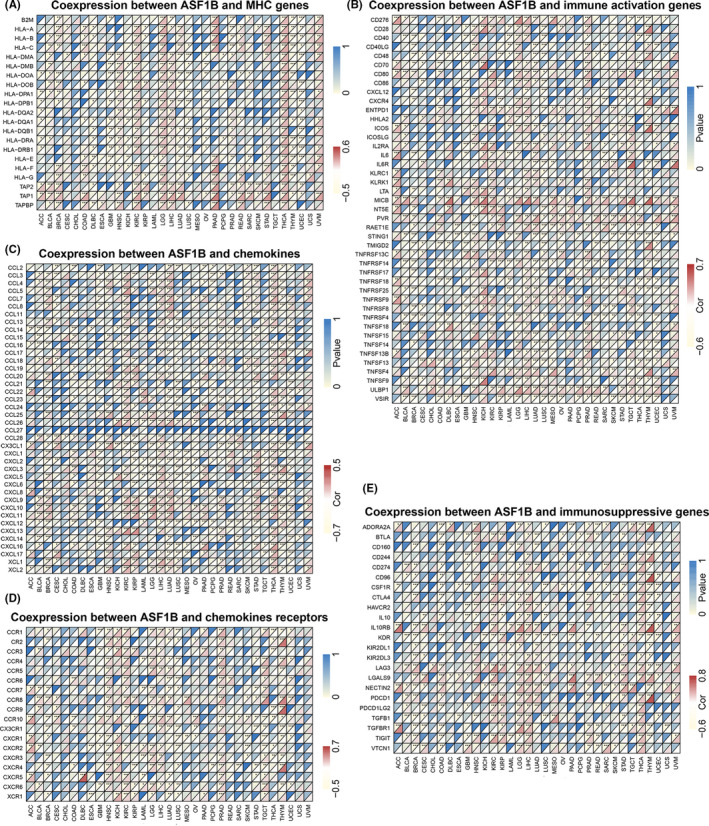
Coexpression of ASF1B with immune‐associated genes in 33 cancer types. (A) Coexpression of ASF1B with MHC genes. (B) Coexpression between ASF1B and immune activation genes. (C) Coexpression of ASF1B with chemokines. (D) Coexpression of ASF1B with chemokine receptors. (E) Coexpression between ASF1B and immunosuppressive genes. *p < 0.05, **p < 0.01, ***p < 0.001

Gene coexpression analyses were further conducted to investigate correlations between ASF1B expression and immune‐related genes in 33 types of cancer. The genes analyzed included those encoding immune activation, immune suppression, major histocompatibility complex (MHC), chemokine, and chemokine receptor proteins. Heat map results showed that nearly all immune‐associated genes except CCL27 were coexpressed with ASF1B and that the major immune‐related genes were positively correlated with ASF1B in HNSC, KIRC, KIRP, LGG, LIHC, and THCA (Figure 12). We also found that the MHC genes were coexpressed with ASF1B in almost all cancer types (besides CESC, CHOL, ESCA, LAML, MESO, PCPG, RGRC, OV, and UCS), particularly in HNSC, KIRC, LGG, LIHC, LUAD LUSC, THCA, THYM, UCEC, and UVM (Figure 12A). In addition, immune activation genes and immunosuppressive genes were coexpressed with ASF1B in all cancer types, though the correlations were relatively small in CHOL, ESCA, PCPG, and USC (Figure 12B, E).

**FIGURE 13:**
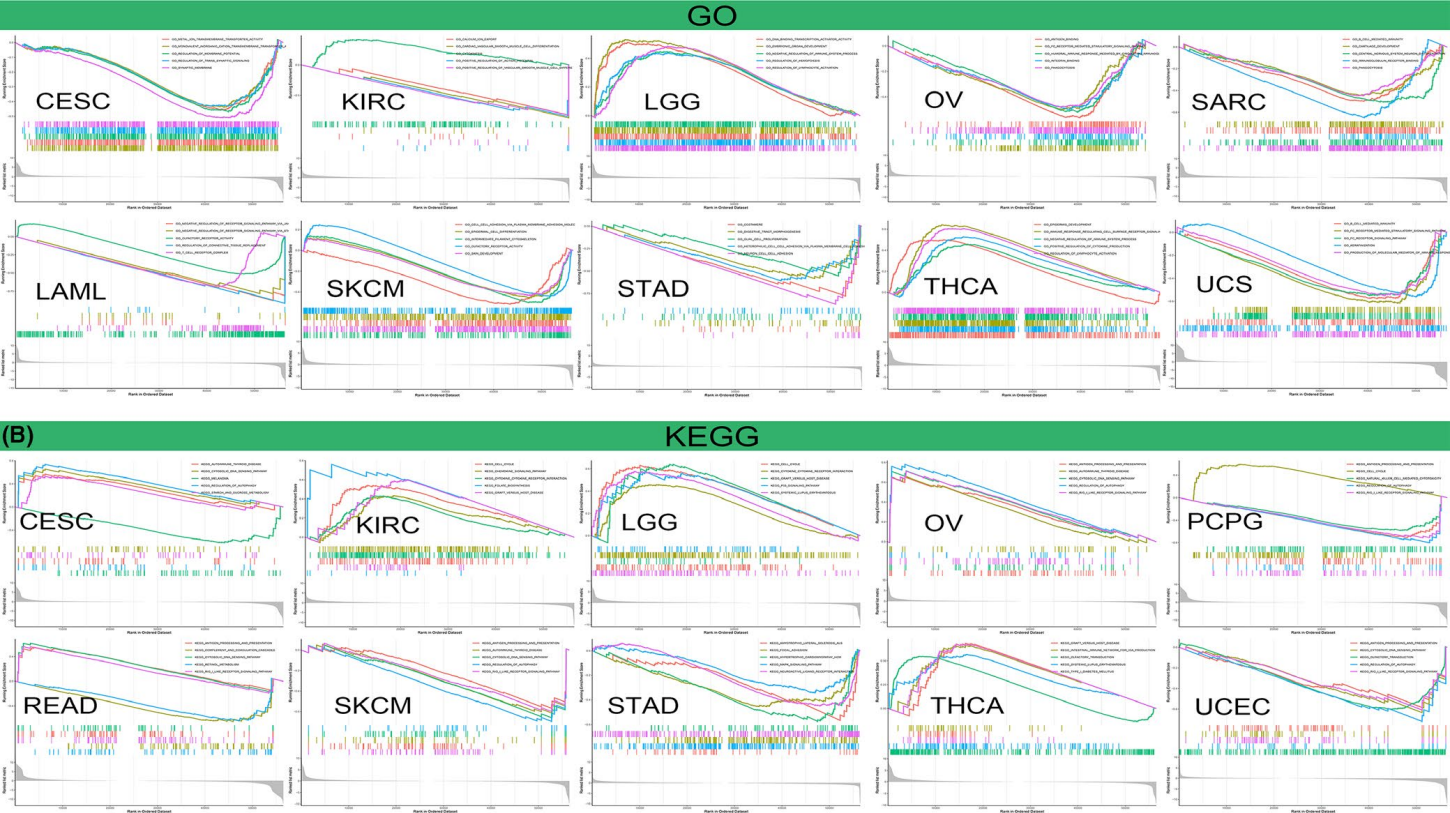
ASF1B pathway analysis in various cancers. (A) GO functional annotations associated with ASF1B in cervical cancer (CESC), kidney clear cell carcinoma (KIRC), lower grade glioma (LGG), ovarian cancer (OV), sarcoma (SARC), acute myeloid leukemia (LAML), skin cutaneous melanoma (SKCM), stomach cancer (STAD), thyroid cancer (THCA), and uterine carcinosarcoma (UCS). (B) KEGG pathways associated with ASF1B in CESC, KIRC, LGG, OV, (pheochromocytoma & paraganglioma) PCPG, rectal cancer (READ), SKCM, STAD, THCA, endometrioid cancer (UCEC). Curves of different colors indicate that ASF1B regulates distinct functions or pathways in various tumors. Upward‐facing curve peaks indicate positive regulation, and downward‐facing curve peaks represent negative regulation

Next, we analyzed GO functional annotations and KEGG pathways associated with ASF1B in various cancers (Figure 13; Figure S4). The data indicated that ASF1B was associated with negative regulation of some immune‐associated functions in OV, including antigen binding, humoral immune response mediated by circulating immunoglobulin, and immune response regulating cell surface receptor signaling (Figure 13A). In SARC, ASF1B was also associated with negative regulation of immune‐related functions, including B cell‐mediated immunity, immunoglobulin receptor binding, and phagocytosis (Figure 13A). KEGG pathway analyses also demonstrated that ASF1B could negatively modulate several key immune‐related pathways, including those involved in antigen processing and presentation, natural killer cell‐mediated cytotoxicity, regulation of autophagy, and RIG‐I‐like receptor signaling in PCPG; antigen processing and presentation, autoimmune thyroid disease, regulation of autophagy, and RIG‐I‐like receptor signaling in SKCM; and antigen processing and presentation, regulation of autophagy, and RIG‐I‐like receptor signaling in UCEC (Figure 13B). In addition to immune‐related pathways, our results suggested that ASF1B also regulates many other pathways, such as those involved in cytosolic DNA sensing, starch and sucrose metabolism, melanoma, phagocytosis, and central nervous system neuron differentiation.

## 4 DISCUSSION

The present study demonstrated that the ASF1B gene was highly expressed in 20 cancers; immunohistochemical (IHC) results supported this trend at the protein level. Our findings in CESC, BRCA, and LUAD resemble the results of previous studies.^4^, ^11^, ^26^ In addition, we identified high ASF1B expression in DLBC and OV, though the TCGA expression data from normal tissues were insufficient. IHC analyses using HPA revealed that ASF1B is undetectable in the ovary and skin, encouraging speculation that ASF1B is expressed at a higher level in DLBC and OV than in normal tissues. Overall, ASF1B is highly expressed in at least 22 types of cancers and high ASF1B expression may be a predictor of tumorigenesis. Furthermore, analyses using HPA demonstrated that ASF1B mRNA is enriched in granulocytes, tonsils, and lymph nodes, all of which play vital roles in the immune system. Therefore, we hypothesize that ASF1B may play a role in cancer development by regulating immune system‐related functions.

Kaplan–Meier survival analyses revealed that high ASF1B expression is associated with poor prognosis in several cancers. A previous study revealed that the upregulation of ASF1B promotes the proliferation of CESCs by forming a stable complex with CDK9.^4^ Additionally, overexpression of ASF1B promoted the proliferation and metastasis of BRCA and LUAD cells and had prognostic value.^11^, ^26^ In the present report, we identified for the first time that high ASF1B expression is also associated with poor prognosis in ACC, KIRC, KIRP, LGG, LIHC, MESO, PAAD, and UVM. Conversely, the high ASF1B expression is predictive of better prognosis in patients with CESC, GBM, LUSC, SKCM, STAD, and THYM, though this may be due to the limited number of samples.

Moreover, we found that ASF1B expression is associated with age in some cancers. ASF1B expression was lower in younger patients with BRCA, CHOL, ESCA, LIHC, LUAD, LUSC, and THYM than in older individuals, while higher ASF1B expression was detected in younger patients with LGG and PRAD. These discoveries may have implications for the selection of immunotherapy therapy regimens for patients of different ages. Our study also found that in most tumors, ASF1B expression correlated with tumor stage; it was particularly relevant in distinguishing between stage I and stage IV tumors. In patients with ACC, BLCA, BRCA, ESCA, KICH, KIRC, KIRP, LIHC, LUAD, LUSC, and UVM, ASF1B expression was higher in stage IV tumors than in stage I tumors. These results clearly suggest that ASF1B can be used as a prognostic biomarker in multiple cancers.

Previous studies have shown that TMB reflects the overall neoantigen load, thus influencing the efficacy of immunotherapy.^27^, ^28^ Furthermore, TMB may be useful as a prospective pan‐cancer predictive biomarker, providing guidance for immunotherapy selection in the age of precision medicine.^29^ It has also been revealed that TMB is correlated with clinical ICI response and that higher TMB is associated with better overall survival.^30^ MSI is also a key biological marker of ICI response. High‐frequency MSI in COAD correlates with increased sensitivity of COAD cells to ICIs, which is predictive of better clinical features and improved prognosis.^31^ This study showed that ASF1B expression is correlated with TMB in 21 cancers and with MSI in 7 cancers. These findings may indicate that ASF1B expression affects the TMB and MSI of various tumors and thus the patient's response to ICI therapy. This promises to inform prognosis and response to immunotherapy in various types of cancer. Based on both previous studies and our own findings, we hypothesize that, in tumor types where ASF1B expression is positively correlated with TMB, high ASF1B expression, and high expression of TMB and MSI predict better prognosis and response to ICI treatment.

TME characteristics can affect the sensitivity of tumor cells to immunotherapy and influence clinical results.^14^ Our findings show that ASF1B plays a vital role in cancer immunity. We performed pan‐cancer transcriptome analyses using TCGA data and found that ASF1B expression is significantly negatively associated with immune components of the TME in GBM and UCEC and negatively associated with stromal components of the TME in eight cancers, including BLCA, COAD, HNSC, LIHC, LUAD, OV, PAAD, and UCEC. The relationship between the proportion of stromal or immune cells in tumors and ASF1B expression can be roughly evaluated by stromal scores or immune scores. Our results further clarify the broad oncologic applicability of ASF1B and confirmed that, in other cancers, the expression of ASF1B is closely associated with the biological activation of various immune cells and immune‐related cytokines. It is well known that cancer cells are kept in check by immune cells, and indeed, a tumor will only develop and progress when the immune cells fail to destroy the preneoplastic cells.^32^ We investigated the relationship among ASF1B expression and immune cell infiltration and prognosis. The results further indicated that ASF1B is negatively correlated with the prognosis of patients with LGG, KIRP, LUAD, MESO, and LUVM, and positively correlated with the prognosis of patients with CESC, SKCM, THYM, and STAD. Therefore, high ASF1B expression in certain cancers leads to decreased immune scores and may result in poor patient prognosis. Additionally, we found that ASF1B is coexpressed with genes encoding MHC proteins, immune suppressors, immune activators, chemokines, and chemokine receptors. Our results suggest that ASF1B expression is closely correlated with tumor immune infiltration and therefore affects patient prognosis and provides a new immunotherapeutic target for the treatment of patients with various types of tumors.

At present, few studies have reported on the immunological role of ASF1B in cancer, and it is generally believed that ASF1B is a promoter of cell proliferation that can also act on the cell cycle.^4^ High ASF1B expression has been shown to affect ccRCC tumor staging and tumor grading via the AKT/P70 S6K1 pathway, leading to poor prognosis. Notably, S6K1 was found to affect the expression of immune response genes.^9^ Our enrichment analyses demonstrate for the first time that ASF1B may affect the etiology or pathogenesis of cancer by functioning in immune‐related pathways, including those involved in antigen processing and presentation, natural killer cell‐mediated cytotoxicity, regulation of autophagy, autoimmune thyroid disease, and RIG‐I‐like receptor signaling pathway.

In conclusion, our first pan‐cancer analysis of ASF1B shows that this gene is highly expressed in most tumor tissues compared to normal tissues and reveals an association between ASF1B expression and clinical prognosis. Our results also suggest that ASF1B may be an independent prognostic factor in multiple cancers and that high ASF1B expression is related to poor prognosis in major tumor types. However, the specific role of ASF1B in each tumor type should be further investigated. Furthermore, ASF1B expression is associated with TMB, MSI, and immune cell infiltration in various cancers. Its effect on tumor immunity varies depending on the cancer type. These discoveries may contribute to clarifying the role of ASF1B in tumor development and provide new regulatory targets for more precise and personalized immune antitumor strategies.

## CONFLICTS OF INTEREST

The authors declare that there are no conflicts of interest.

## AUTHOR CONTRIBUTIONS

Ximing Xu and Xiaoqin He designed this study. Hua Zhu and Xiaoyu Zhang analyzed the data. Hua Zhu and Xinyao Hu wrote the article. All authors read and approved the final manuscript.

## ETHICS APPROVAL AND CONSENT TO PARTICIPATE

This study was approved by the Ethics Committee of Renmin Hospital of Wuhan University.

## Supporting information

Figure S1

Figure S2

Figure S3

Figure S4

Table S1

Table S2

## Data Availability

The datasets generated and/or analyzed during the current study are available from the corresponding author upon reasonable request in compliance with ethical standards.
