# Supplementary figures and images for: Comprehensive analysis of pan‐cancer reveals potential of ASF1B as a prognostic and immunological biomarker
Source: Cancer Med. 2021 Sep 2;10(19):6897–916. doi: 10.1002/cam4.4203 (PMC8495294; doi:10.1002/cam4.4203)

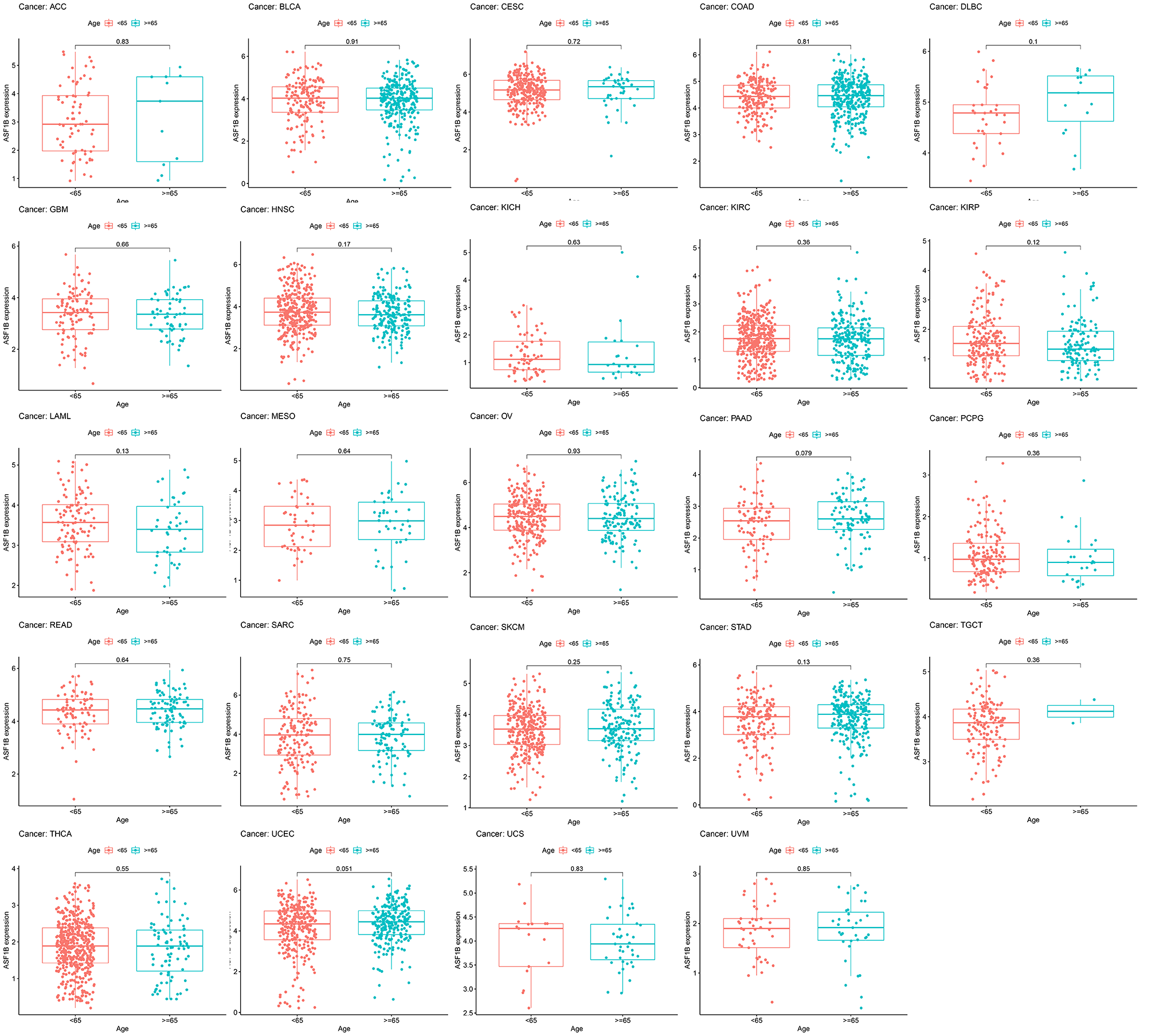

Supplement: Supplementary file 1 — Figure S1 [file CAM4-10-6897-s005.tif]

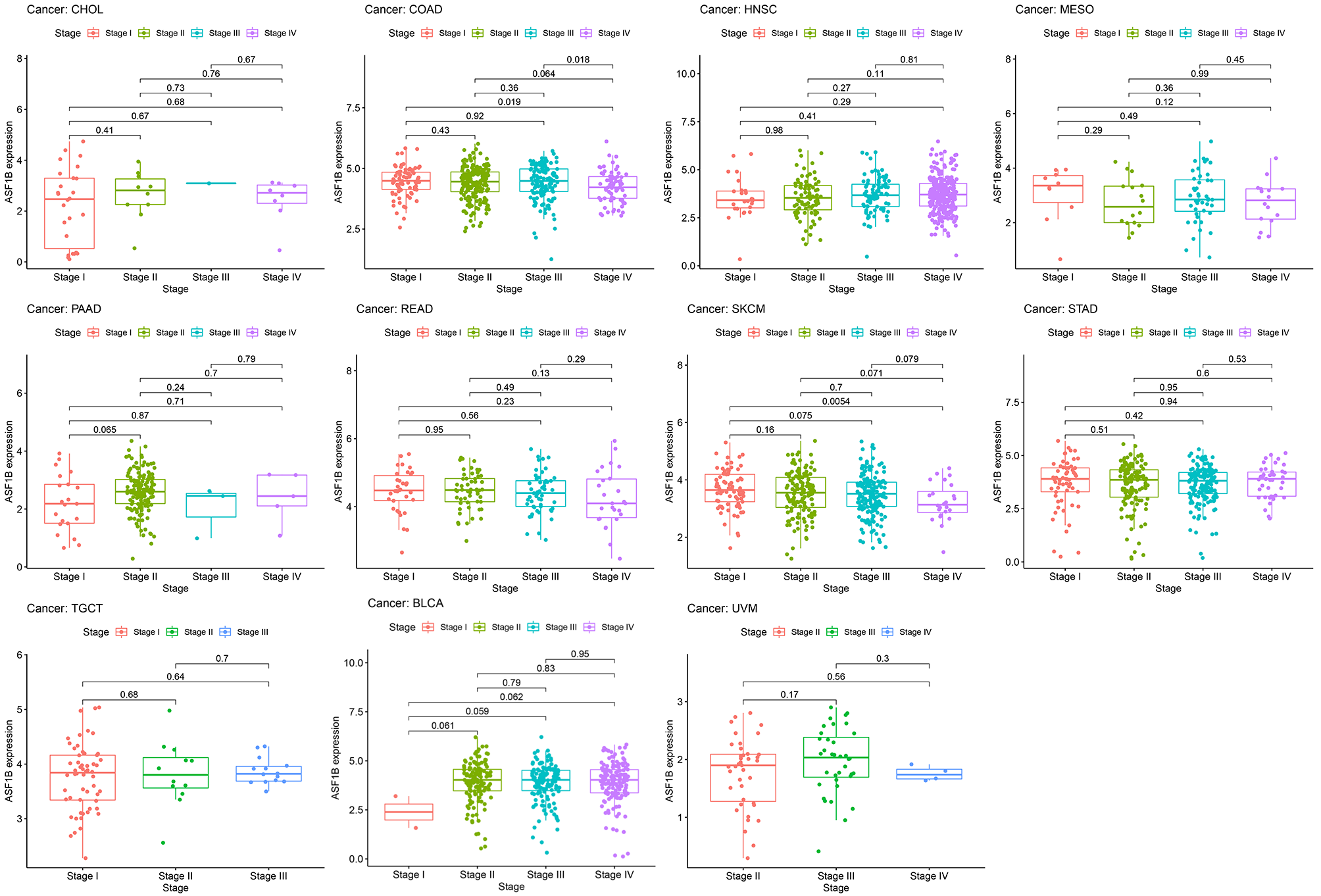

Supplement: Supplementary file 2 — Figure S2 [file CAM4-10-6897-s002.tif]

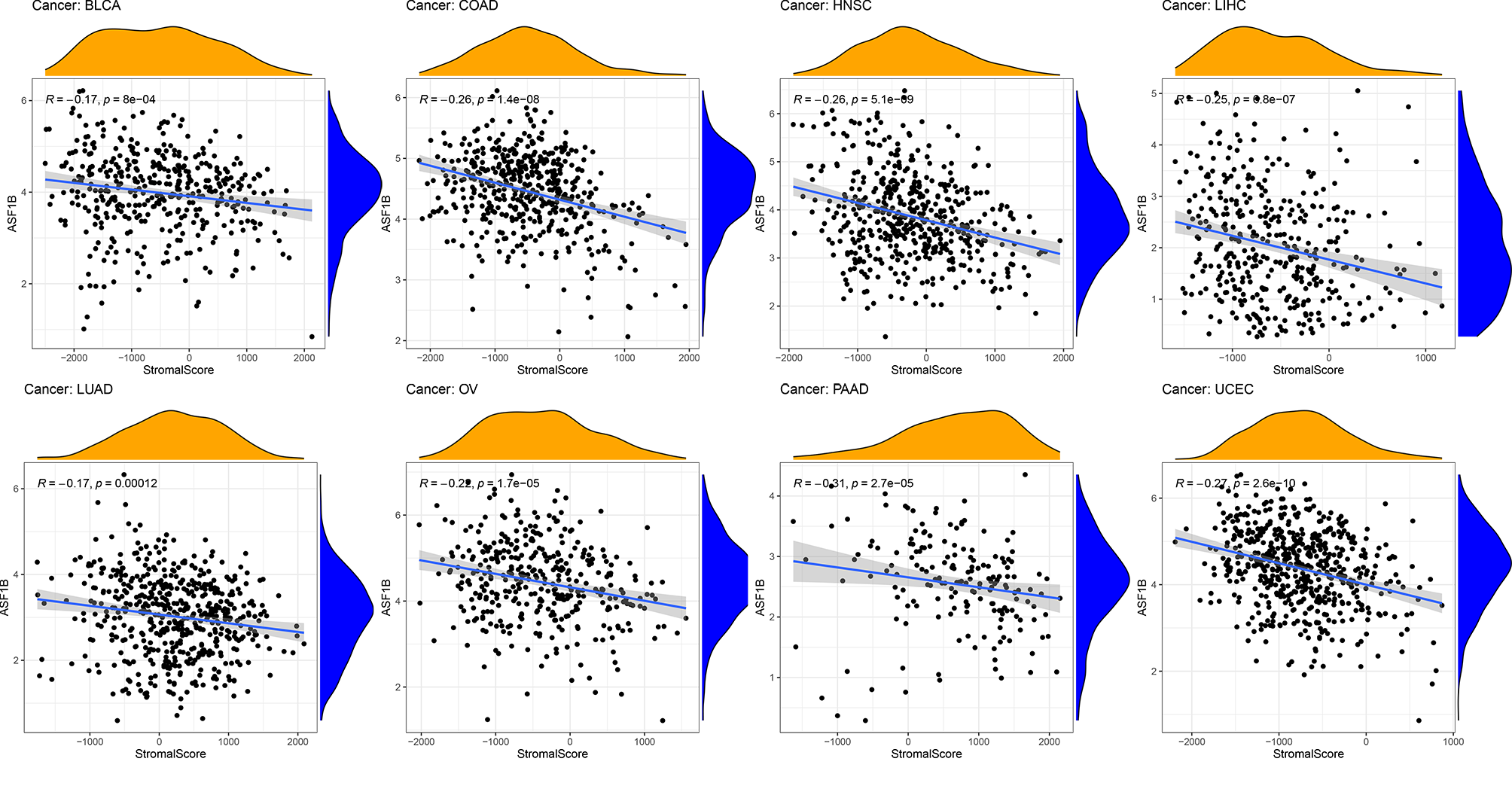

Supplement: Supplementary file 3 — Figure S3 [file CAM4-10-6897-s006.tif]

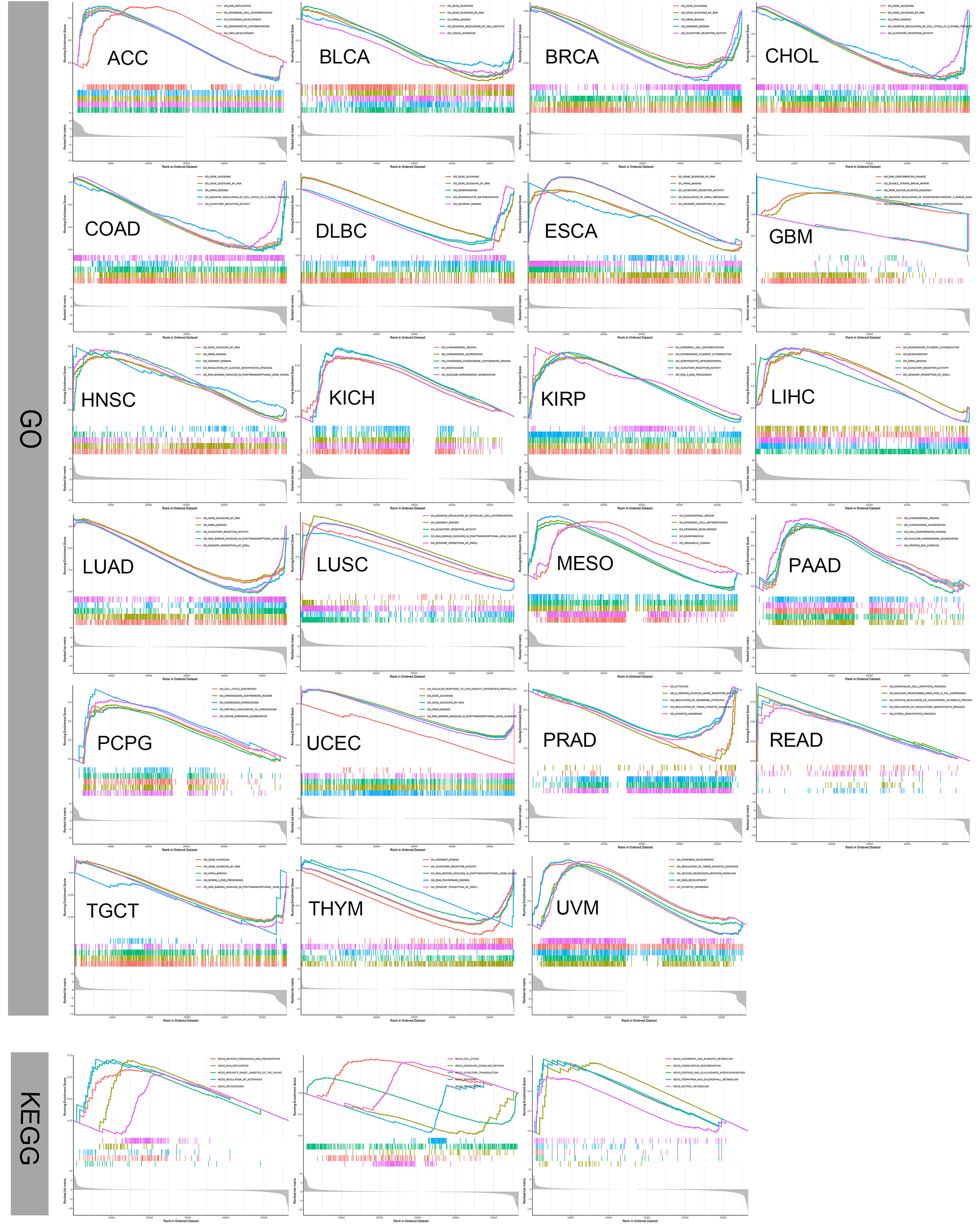

Supplement: Supplementary file 4 — Figure S4 [file CAM4-10-6897-s004.tif]
