# Supplementary material for: Comprehensive analysis of pan‐cancer reveals potential of ASF1B as a prognostic and immunological biomarker
Source: Cancer Med. 2021 Sep 2;10(19):6897–916. doi: 10.1002/cam4.4203 (PMC8495294; doi:10.1002/cam4.4203)
Supplement: Supplementary file 5 — Table S1 [file CAM4-10-6897-s001.docx]

**Supplement Table 1.** The detailed information of 33 cancers.

| **Abbreviation** | **Full name** | **Number of normal samples** | **Number of cancer samples** |
| --- | --- | --- | --- |
| ACC | adrenocortical carcinoma | 0 | 79 |
| BLCA | bladder Urothelial Carcinoma | 19 | 411 |
| BRCA | breast invasive carcinoma | 113 | 1104 |
| CESC | cervical squamous cell carcinoma and endocervical adenocarcinoma | 3 | 306 |
| CHOL | cholangiocarcinoma | 9 | 36 |
| COAD | colon adenocarcinoma | 41 | 471 |
| DLBC | lymphoid Neoplasm Diffuse Large B-cell Lymphoma | 0 | 48 |
| ESCA | esophageal carcinoma | 11 | 162 |
| GBM | glioblastoma multiforme | 5 | 168 |
| HNSC | head and Neck squamous  cell carcinoma | 44 | 502 |
| KICH | kidney Chromophobe | 24 | 65 |
| KIRC | kidney renal clear cell carcinoma | 72 | 535 |
| KIRP | kidney renal papillary  cell carcinoma | 32 | 289 |
| LAML | acute Myeloid Leukemia | 0 | 151 |
| LGG | brain lower grade glioma | 0 | 529 |
| LIHC | liver hepatocellular carcinoma | 50 | 374 |
| LUAD | lung adenocarcinoma | 59 | 526 |
| LUSC | lung squamous cell carcinoma | 49 | 501 |
| MESO | mesothelioma | 0 | 86 |
| OV | ovarian cancer | 0 | 379 |
| PAAD | pancreatic adenocarcinoma | 4 | 178 |
| PCPG | pheochromocytoma and paraganglioma | 3 | 183 |
| PRAD | prostate adenocarcinoma | 52 | 499 |
| READ | rectum adenocarcinoma | 10 | 167 |
| SARC | sarcoma | 2 | 263 |
| SKCM | skin cutaneous melanoma | 1 | 471 |
| STAD | stomach adenocarcinoma | 32 | 375 |
| TGCT | testicular Germ Cell Tumors | 0 | 156 |
| THCA | thyroid carcinoma | 58 | 510 |
| THYM | thymoma | 2 | 119 |
| UCEC | uterine corpus endometrial carcinoma | 35 | 548 |
| UCS | uterine Carcinosarcoma | 0 | 56 |
| UVM | uveal melanoma | 0 | 80 |
|  |  | 730 | 10327 |
